# An Unusual Localization of Lunate in a Transcaphoid Volar Lunate Dislocation: Current Concepts

**DOI:** 10.1155/2019/7207856

**Published:** 2019-07-11

**Authors:** Grigorios Kastanis, George Velivasakis, Anna Pantouvaki, Manolis Spyrantis

**Affiliations:** ^1^Department of Orthopaedics, General Hospital of Heraklion-Venizeleio, Crete, Greece; ^2^Department of Physiotherapy, General Hospital of Heraklion-Venizeleio, Crete, Greece

## Abstract

Perilunate dislocation and fracture dislocations are rare injuries corresponding to 10% of all carpal injuries. They usually come with high-energy trauma, with associated injuries representing 61%. Volar lunate dislocation or fracture-dislocation accounts for 3% of perilunate injuries. We present a case of a 42-year-old polytrauma male, transmitted to our department 48 hours after a car accident with a trans-scaphoid volar lunate dislocation. During operation, the lunate was displaced volarly to the ulnar side of the wrist, forward to the styloid process of the distal ulna, while the scaphoid fracture appeared at the waist with comminution, and the proximal pole of the scaphoid protruded under the dorsal capsule. Carpal injuries are often missed out in polytrauma patients, and these injuries are underestimated because of the severity of the other visceral or extremity lesions. Untreated or improperly treated, those injuries lead to serious morbidity and loss of function. Therefore, good functional prognosis with decreased percentage of complications can be achieved following early recognition and early open surgical ligamentous complex repair.

## 1. Introduction

Perilunate fracture dislocation and lunate dislocation are a combination of injuries (ligament-bone) around the lunate. This type of injury is rare and accounts for 10% of all wrist injuries [1]. These traumatic lesions represent a broad spectrum in which the trans-scaphoid dorsal perilunate fracture dislocation appears more frequently [2]. Trans-scaphoid volar dislocation of the lunate with volar displacement of the lunate into the radiocarpal joint or into the distal forearm is extremely rare [3]. This is a hyperextended high-energy wrist injury, with an extensive ligamentous disruption, which is unstable and requires open reduction and internal fixation [4]. Early recognition of this injury and restoration of carpal malalignment is very important to avoid significant complications such as median nerve injury, carpal instability, avascular necrosis of the lunate, complex regional syndrome, unreliable return of function, and posttraumatic arthritis requiring a secondary salvage surgical procedure.

## 2. Case Report

**Figure 1:**
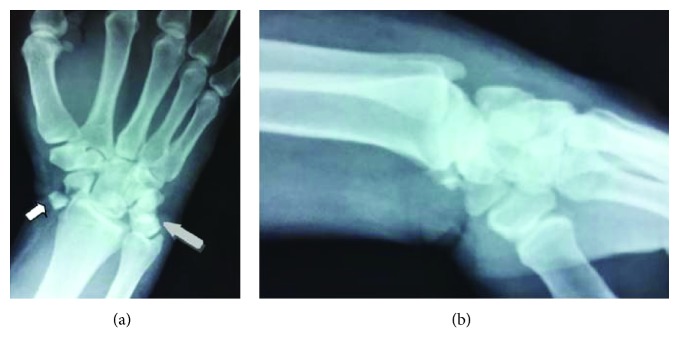
Preoperative X-rays of the left wrist (grey arrow shows the lunate, white arrow shows the scaphoid fracture).

**Figure 2:**
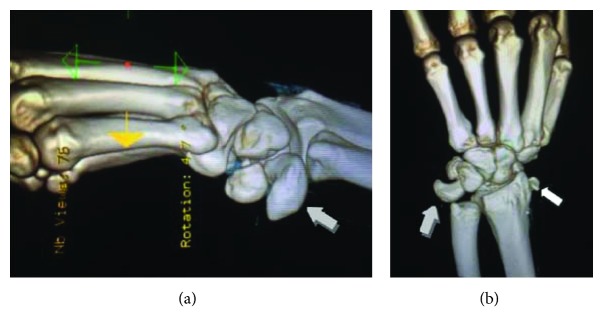
Preoperative 3D CT/scan of the left wrist showing trans-scaphoid volar dislocation of the lunate (grey arrow shows the dislocation of the lunate and the white arrow the proximal pole of the scaphoid).

**Figure 3:**
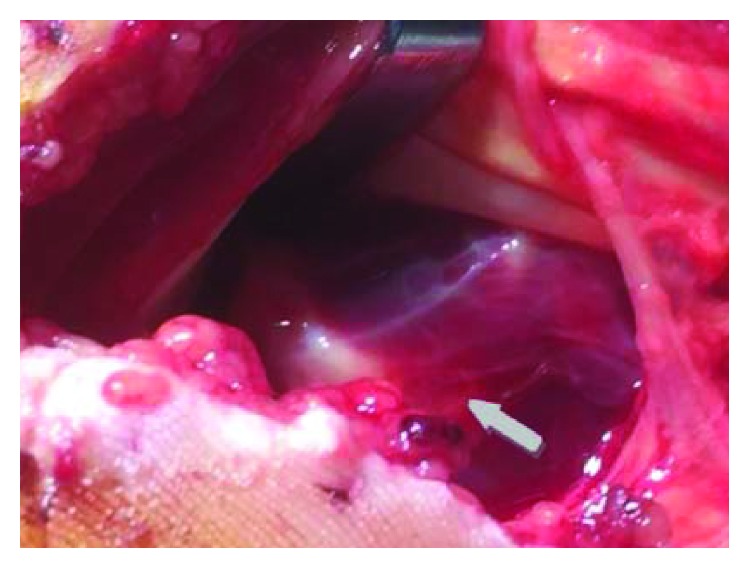
Palmar view of the dislocated lunate (grey arrow).

**Figure 4:**
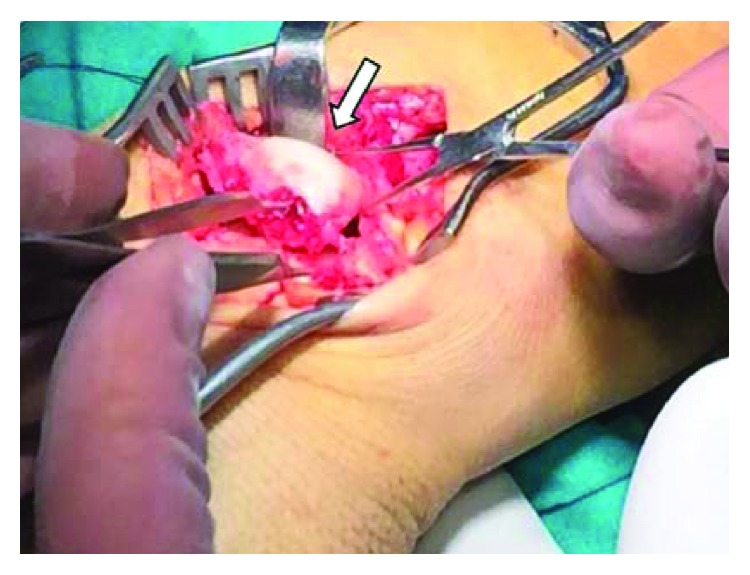
Dorsal view proximal pole scaphoid (white arrow).

**Figure 5:**
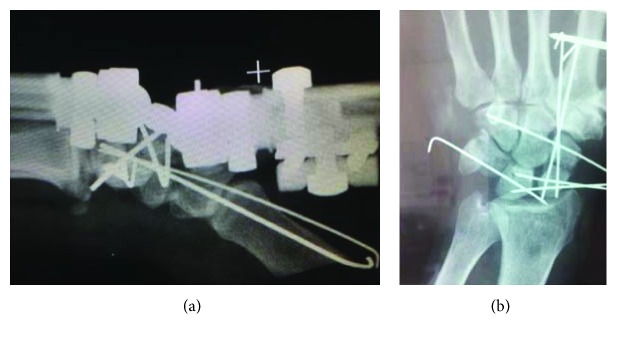
Immediate postoperative X-rays of the left wrist showing open reduction and internal fixation with Kirschner wires and external fixation.

A 42-year-old polytrauma male was transferred from another medical union to our department 48 hours after a car accident with multiple injuries. He had multiple rib fractures (4th, 5th, and 6th ribs on the right side of the chest) and rupture grade III of the right kidney. Radiography examination showed a trans-scaphoid volar lunate dislocation to his left wrist, which was the dominant hand (Figures 1(a) and 1(b)). At the clinical examination, the patient had the left wrist swollen and reported numbness in the distribution of the median nerve. Hand circulation was not jeopardized. Computed tomography examinations revealed a dorsal trans-scaphoid fracture and volar dislocation of the lunate (Figures 2(a) and 2(b)). The lunate was localized in the palmar side of the left wrist, forward to the styloid process of the distal ulna. The patient was taken to the operating theatre where, under general anesthesia and tourniquet, an extended carpal tunnel approach was performed, in which the transverse carpal ligament and forearm fascia were released and the lunate was relocated in the radiocarpal joint (Figure 3). The volar ligamento-capsular complex (radiocarpal-ulnacarpal ligaments) was ruptured and restored with nonabsorbable sutures. A dorsal approach over the Lister tubercle between the 3rd and 4th extensor compartments was used to achieve the reduction and to fixate the bone injuries. Continuing the approach to expose the dorsal surface of the wrist bone, a trapezium flap of the dorsal wrist capsule was elevated from the radial side to the apex of the triquetrum. A scaphoid waist comminuted fracture appeared with the proximal pole of the scaphoid protruding under the dorsal capsule (Figure 4). The fixation of the scaphoid fracture was done with two 1.4 mm Kirschner wires from the dorsal to the volar direction. No bone grafting was used. The lunate was cleared out from all ligament attachments. The scapholunate interosseous ligament (SLIL) was disrupted, and only a residue of this was attached on the proximal pole of the scaphoid. The SLIL was repaired with an anchor suture, and three 1.4 mm Kirschner wires stabilized the joints (two from the scaphoid to the lunate, one from the scaphoid to the capitate) to support ligamentous repair. The lunotriquetral ligaments appeared completely torn and were fixed with an anchor suture and one 1.4 mm Kirschner wire from the triquetrum to the lunate. Finally, an external fixator (Penning Dynamic Wrist Fixator) was performed as a neutralization frame. Carpal alignment and k-wire position were confirmed with a C-arm intraoperatively and X-rays postoperatively (Figures 5(a) and 5(b)). Postoperative median nerve symptoms resolved after 10 days. Two weeks after surgery, the sutures were removed. The patient followed a rehabilitation program of both passive-assisted and active exercises initiated in the digits, preventing finger stiffness and reducing edema. After 8 weeks, the external fixator and Kirschner wires were removed and the patient began the second stage of rehabilitation. At this phase, friction massage (scar tissue treatment) and manual therapy as well as C.P.M. (continuous passive motion) were performed in each session. The patient was instructed precisely and was given a regime of active and strengthening exercises. Hand sensitivity training was considered as well.

**Figure 6:**
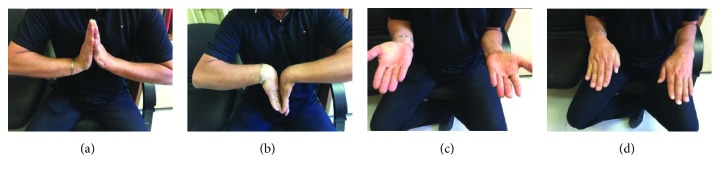
Range of motion at one year.

Four months after surgery, the patient returned to the previous functional activity (manual worker) with a pain-free wrist. Finally, at one-year follow-up, the patient remained asymptomatic and the range of motion (measured with a handheld goniometer) was as follows: active wrist extension 40°/44°, flexion 60°/65°, radial deviation 15°/17°, ulnar deviation 33°/30°, and full range of pronation-supination of the forearm (Figures 6(a)–6(d)). The grip strength (measured with a Jamar dynamometer) averaged 42 lbs/54 lbs compared to the contralateral hand. The functional score according to the Mayo Wrist Score was 80, to VAS was 0, and to the QuickDASH score was 9,1, which were excellent results in relation with the contralateral wrist.

## 3. Discussion

Trans-scaphoid volar dislocation of the lunate injuries belongs to the greater arc injury in which the lunate gradually rotates invading to the carpal canal. Green and O'Brien (1978) first describe a variation of this injury in which the lunate was palmary dislocated in the radiocarpal joint together with the proximal pole of the fractured scaphoid. Until today, this pattern of injury in international literature has been reported in eight cases [5]. Al Khayarin et al. [6] describe a case with trans-scaphoid volar dislocation of the lunate with displacement into the palmar side of the distal forearm, while Koh et al. [3] describe a case with trans-scaphoid volar lunate dislocation combined with complete scapholunate dissociation and total extrusion of the soft tissue localized in the palmar side of the forearm. The current case is unique because the lunate was dislocated into the volar aspect of the wrist forward in the styloid process of the distal ulna (detached from all ligament attachments) while the proximal pole of the scaphoid fracture was dorsally displaced.

It is generally accepted according to the severity of perilunate fracture dislocations that the gold standard is surgical treatment and only the approach remains controversial [3]. There is a proponent of combining the double approach with all the advantages of the method (release median nerve, restore palmar ligament, and dorsal fixation of carpal bone fractures) and the proponent of a single isolate dorsal approach. Herzberg [7] suggested the single dorsal approach in cases in which the rotation of the dislocated lunate is less than 90° and the double approach when the rotation of the bone is more than 90°. In our case, we performed the double approach, and through the palmar one, we relocated the dislocated lunate [8, 9].

Among the surgical procedures of the scaphoid fracture, there are two modalities of implant: cannulated screw or Kirschner wire. In the majority of the cases, it has been reported that the fracture is in the middle third and was comminuted [10, 11]. In this situation, the osteosynthesis of the fracture with the Kirschner wires is preferable because it avoids the risk of rotational deformity [8]. Postoperative immobilization is necessary to avoid the loss of reduction (external fixation or short arm cast) for a period of 6-12 weeks [12, 13].

**Figure 7:**
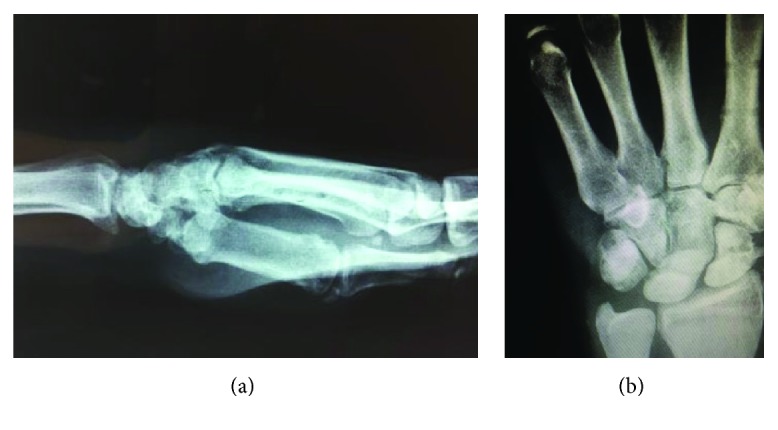
X-ray at one year.

A common complication is osteonecrosis of the lunate or of the proximal pole of the scaphoid. Gellmann et al. [14] describe that the avascular change of the lunate after dislocation may be transient and a possibility of revascularization could exist. Ekerot [15] argues that revascularization of the lunate can be repaired by the unit scaphoid fracture or the intact scapholunate interosseous ligament. Functional outcomes after such injuries are variable. Massoud and Naam [16] refer that lesser arc injuries have poorer outcomes than greater arc injuries while Kremmer et al. [17] suggested that the functional outcomes after these injuries deteriorate with time. Forli et al. [18] in a retrospective study of ten years found that while radiographic imaging of posttraumatic arthritis was present, the functional outcomes were not affected. In our patient, the lunate was disrupted from the attachments of all ligaments and had significant displacement. At one year follow-up, there is no sign of osteonecrosis of the lunate or the scaphoid, but perhaps a longer period of time is required in order to evaluate if the usual complications will develop (Figures 7(a) and 7(b)).

## 4. Conclusion

Trans-scaphoid volar lunate dislocation is a very rare and high-energy injury. A large percentage of these carpal lesions is often missed out at the initial evaluation of the patients. Missing or improperly treating these injuries leads to serious morbidity and loss of function. Therefore, good functional outcomes can be achieved following early recognition of the injuries, repair of the capsuloligamentous injuries, and restoration of carpal alignment.
